# Influences of Differing Menarche Status on Motor Capabilities of Girls, 13 To 16 Years: A Two-Year Follow-Up Study

**DOI:** 10.3390/ijerph18115539

**Published:** 2021-05-22

**Authors:** Barry Gerber, Anita E. Pienaar, Ankebe Kruger

**Affiliations:** 1 Physical Activity, Sport and Recreation (PhASRec), School of Human Movement Sciences, Faculty of Health Sciences, Potchefstroom Campus, North-West University, Private Bag X6001, Potchefstroom 2520, South Africa; anita.pienaar@nwu.ac.za; 2 Centre of Health and Human Performance (CHHP), Faculty of Health Sciences, Potchefstroom Campus, Institute for Psychology and Wellbeing (IPW), North-West University, Private Bag X6001, Potchefstroom 2520, South Africa; ankebe.kruger@nwu.ac.za

**Keywords:** growth, longitudinal, menarche, motor capabilities

## Abstract

Puberty and the onset of menarche influences the motor performance of girls. However, the magnitude of these influences during varying maturity status, is not clear. This longitudinal study over two years aimed to investigate differences in motor fitness between early and late developing girls based on pre- and post-menarche status. A convenience sample (*n* = 58) of girls aged 13.51 ± 3.51, divided by means of the Status Quo method into pre (*n* = 13) and post-menarche (*n* = 45) groups, was used. Motor fitness was tested once annually by standardized protocols. Basic statistics, independent *t*-testing and a repeated measures ANOVA with a post hoc Bonferonni correction were used (*p* < 0.05 = statistical significance). Effect sizes were determined by Cohen’s *d*-values. Only explosive upper body strength differed significantly between groups during baseline, favoring post-menarche girls. Initially, post-menarche girls showed advantages in hand-eye coordination and speed (*p* > 0.05) with pre-menarche girls performing better in agility and explosive leg strength (*p* > 0.05). At 15.51 years, no significant, between-group differences were found. Pre-menarche girls surpassed post-menarche girls in hand-eye coordination and 0–40 m speed and post-menarche girls displayed higher explosive leg and upper body strength scores (*p* > 0.05). Our data show that the potential to excel in sport based on motor capabilities can only be accurately estimated 1–2 years after reaching menarche.

## 1. Introduction

Differential timing of the onset of menarche has various consequences for girls [1,2,3,4,5,6,7,8,9,10]. Understanding these effects, especially on the physical and motor abilities of girls, can provide coaches, physical education teachers and others involved in the health and well-being of adolescent girls, with valuable insight into the physiology of young girls, especially the potential effects of early and late exposure to sex hormones on their motor and physical abilities [1,2]. In this regard, researchers [3,4] report that the largest variation in sexual maturity among girls is found between the ages of 12 and 16 years, showing clear associations with anthropometric characteristics and motor fitness [5]. Due to menarche being associated with various physical changes that occur during puberty [6], researchers have described differential age of onset of menarche as a risk factor for a number of adverse health outcomes, including obesity [7], type 2 diabetes [8] and cardio-metabolic traits [9], all mostly associated with girls who reach menarche at a young age (early developing girls). In addition, the timing of the onset of menarche also influences the development of motor and physical fitness capabilities of adolescent girls which has consequences for sport performance [10].

Menarche, which is reached during puberty (11.8 to 13 years), on average at the age of 12.8 years [11,12] is a critical milestone in female’s journey to adulthood, from both a socio-cultural and medical perspectives [13]. Age of menarche, which occurs about 6 months to 1 year after reaching the maximum growth spurt or peak height velocity (PHV) [12,14], is not rigid and is influenced by a variety of factors, such as genetic factors, nutrition and health status [15,16]. The menstrual cycle is comprehended as a biofeedback system; therefore, each structure and or gland is affected by the activity of the others [17]. The onset of menarche, in turn, directly affects the physical and motor capabilities of girls which have consequences, specifically, for those who participate in sport [11]. The time period of puberty induces critical morpho-functional transitions across the adolescence period [18]. These changes influence anthropometric characteristics and body composition (height, weight, body fat and muscle mass) which again affects motor performance (speed, agility, explosive power, hand-eye coordination and muscle strength) of girls [11,19] and subsequently, sport performance [11,20,21,22,23,24]. More specifically, speed, agility and explosive power are directly influenced by body dimensions such as sitting height and arm span [11].

The literature, however, indicates that biological maturity is not the only role player in girls’ motor development, as biological maturity for example only accounts for approximately 2.8% of variation in girls’ coordination. Literature also indicates that anaerobic improvement still occurs after puberty indicating that biological maturity plays a significant role in speed [11]. In this regard, peak development in muscle strength and endurance, which is directly related to speed and agility, commences approximately 0.5–1 years after reaching PHV [11].

Various studies that focused on changes in girl’s motor and physical capabilities during adolescence have been conducted globally [25,26,27,28,29,30,31,32,33,34,35,36] although to a lesser extent in South Africa [22,27,32,33,34,35,36,37,38,39,40]. Results from the above studies concluded that motor capabilities are influenced by biological maturity, although neurological development and fitness and sport training also showed influences. However, biological maturity differences related to the age of onset of menarche were not the main differential factor in these analyses. Furthermore, contradictory results regarding the influence of menarche on girl’s motor capabilities are also found, where some research [11] reports no correlations between peak motor development before, during and after menarche in relation to the onset of menarche, while others found [22] significant differences between early, middle and late developing girls. Results of the studies that were reviewed indicated that physical and motor fitness are influenced in different ways by biological maturation during puberty. Most of these studies are older than 10 years old, which questions the relevance of the findings for modern populations. In addition, limited studies analyzed influences of varying menarche status (or the phenomena of early and late development) on motor fitness longitudinally. This background identifies gaps in the literature regarding the understanding of pathways of change in motor fitness as a result of the interrelatedness of different human systems, more specifically, how the differential timing of menarche status will affect the motor fitness capabilities of early and late developing girls.

Consequently, as it can take up to ten years to reach elite performance in a particular sport [41] and as late menarche occurrence seems to favor athletic performance [22,42] it is important to understand developmental motor fitness pathways in girls of differing maturity status especially during the mid to late adolescence period. Such knowledge can aid the process of identifying girls with true sporting potential during this time period or earlier to ensure optimal development. Furthermore, knowledge in this regard will aid coaches and trainers to address maturational differences to the benefit of all girls of similar chronological ages. This can aid the implementation of physiologically sound long-term athlete development plans that will address the developmental needs of all girls, especially late maturing girls, who will most probably be the sporting stars in the future. The aim of this study is therefore to investigate the extent of developmental differences in speed, agility, explosive power and coordination in pre (or late developing) and post-menarche (or early developing) girls by studying it over a longitudinal period during the mid-adolescence period. Findings from the study will aid in a better understanding of the influence of the phenomena of menarche on girls’ explosive upper body strength, hand-eye coordination, explosive leg strength, agility and speed development. This understanding can then contribute to the compilation of sport development programs that are aligned with girls’ maturational status. In addition, the results of this study can contribute to a more accurate selection of girls during the mid-adolescent period who have the potential to excel in sport, based on motor specific characteristics.

## 2. Materials and Methods

### 2.1. Research Design

This study is a sub-study of the longitudinal project “Growth and sport psychological characteristics of talented adolescents” that was conducted over a two-year follow-up period spanning a 3-year school period (2010–2012). The data were collected using anthropometric and motor tests as well as questionnaires. Anthropometric measurements took place three times a year (four months apart) with the motor tests measured once annually. Baseline measurements were taken in February 2010 and the last measurements took place in November 2012, resulting in nine time-point measures for growth (T1–9) and three-time point follow-up measures for motor fitness skills (T1, T2, T3). This time period includes girls between the ages of 13 and 16 years of age.

### 2.2. Research Group

The participants were recruited by means of convenience sampling. All grade 8 learners of one quintile 5 school (quintile 1 = low socio-economic quintile, to quintile 5 = high socio-economic quintile) in Potchefstroom in the Northwest Province of South Africa were invited to take part in the study. Since the school had boarding facilities, all grade 8 learners enrolled in 2010 represented 46 different primary schools. In 2010, 95 girls with a mean age of 13.73 + 0.48 years participated in the baseline measurements. The final group, who completed all follow-up measurements in November 2012, consisted of 58 girls with a mean age of 16.27 ± 0.36 years. A power calculation indicated that group sizes of 14 participants are needed to observe medium effects of differences. The loss of subjects to follow-up were 37 girls (38%) due to various reasons including parents of children moving elsewhere, children moving between schools or incomplete datasets.

### 2.3. Ethical Approval

Ethical approval for the execution of the study based on the Declaration of Helsinki was obtained from the Health Research Ethics Committee of the North-West University’s Potchefstroom Campus (NWU 00199-15-A1). Permission for the project was also obtained from the principal of the school involved. Learners who had parental permission and who gave assent themselves were subjected to the testing protocol.

### 2.4. Measuring Instruments

All measurements were conducted by trained researchers from the North-West University. Post graduate students in Human Movement science, specializing in Sport Science and Kinderkinetics assisted in the data collections. These students were trained intensively beforehand on the standardized processes that had to be followed. All of them also had Level 3 certification in Kinatropometry measurements. The principle investigator of the study oversaw all data aspects of the data collection to ensure valid and accurate data at each time point of the follow up period.

#### Age of Menarche

The age of menarche was determined by the Status Quo method [37,43] during the baseline measurements of each year in February in 2010, 2011 and 2012 (T1, T2 and T3). The girls had to indicate on a questionnaire by selecting YES or NO whether they have had their first menstrual cycle on or before the date of testing. If they have answered YES, they also had to indicate the month and year when their first menstrual cycle had started.

A high percentage of the final group (77%, *n* = 45) already reached menarche during the baseline measurement in grade 8 (2010) at a mean age of 13.51 years. For the purpose of this study, the group was divided into a pre-menarche group (*n* = 13) and post-menarche group (*n* = 45) according to the menarche status at baseline. All comparisons were done based on this grouping.

### 2.5. Motor Measurements

The Australian Sport Search Program protocol commonly used in Australia for sports talent identification in children of 12 years and older was used [44]. The test protocol involves six motor tests (beep test, basketball throw, 40 m speed test, 10 m agility test, vertical jump and throw-and-catch-test). All motor tests were conducted according to this protocol. In addition, girls 0–10 m speed were also tested. To obtain more details regarding the methods use, please refer to Gerber et.al [45].

### 2.6. Body Composition

The protocol as developed by the International Society for the Advancement of Kinanthropometry (ISAK) was used for stature and body weight measurements [46]. Stature was measured by using a standardized calibrated stadiometer and body weight was measured by means of an electronically calibrated scale (Omro BF 511). Body weight in kilograms divided by height in meters squared or, BMI = x KG/[y M (×) y M] (x = bodyweight in KG, y = height in M) were used to calculate BMI. Percentage fat mass and muscle mass were measured with an electronically calibrated bio-impedance, body composition apparatus (Omro BF 511). Accuracy of the Omron models differs from model-to-model (+3.5 and 4.1%) based on the Standard Estimation Error (SEE). The SEE indicates that 68% of all measurements for different users are accurate within 3.5–4.1%, relative to body fat percentage (kg/m²). (https://www.omron-healthcare.com/en/products/weightmanagement, accessed on 15 March 2021). Unfortunately, no data were available for baseline muscle and fat mass measurements due to the measurements obtained from the body composition analyzer only being available from the second year of the study as a result of unforeseen circumstances (T2). BMI was, however, calculated and reported for T1 from mass and stature measurements.

### 2.7. Statistical Analysis

The data were analyzed with the “Statistica for Windows 2017” Statsoft computer program [47]. Descriptive characteristics, including means, standard deviations (sd) and minimum and maximum values were calculated. The data were firstly checked for normality. By checking the distributions of the variables, no serious deviations of normality were detected. Independent *t*-testing were used to determine group differences in motor fitness characteristics at each time point with statistical significance set at *p* < 0.05. Effect sizes of these differences were also calculated to determine practical significance of differences using Cohen’s *d*-values with *d* > 0.2 indicating a small effect size, *d* > 0.5 a medium effect size and *d* > 0.8 a large effect size [48].

Repeated measures ANOVA was used to analyze changes in motor skills and capabilities over time followed by a post hoc Bonferonni adjustment to determine the statistical significance of group differences over time as well as the interaction effect over time, with statistical significance set at *p* < 0.05 (*p* < 0.05).

## 3. Results

Table 1 reports the group’s baseline and follow-up descriptive characteristics.

Fifty-eight girls, that included the pre-menarche (*n* = 13, 23%) and post-menarche group (*n* = 45, 77%) completed all measurements. Although the pre- and post-menarche groups as divided at baseline, were used for all analysis, 91% (T2) and 98% (T3) of the group had reached menarche respectively during follow-up measurements. No significant age differences (0.01 years, *p* > 0.05) were found between the groups during baseline measurements, with a mean group age of 13.51 years at baseline.

Table 2 displays the body composition (fat and muscle mass percentage) of the sub-groups during the first and second follow-up measurements. Unfortunately, no data were available for baseline muscle and fat mass measurements due to the body composition analyzer only being introduced during T2. A lower BMI at all three time points and higher muscle mass and lower fat mass were found in the pre-menarche group during T2 and T3. Group differences in fat mass decreased from T2 (4.82%; *p* < 0.05, *d* = 0.68) to 2.71% (*p* > 0.05; *d* = 0.31). Differences in muscle mass of the groups stayed unchanged from T2 to T3. Group differences were also only of small to medium practical significance. BMI differences between groups were borderline statistically significant (*p* = 0.06) during baseline, although became insignificant at T2 and T3. These differences in BMI were of medium practical significance during T1 (*d* = 0.60), but declined to small practical significant differences during T2 (*d* = 0.35) and T3 (*d* = 0.25).

Table 3 shows the descriptive characteristics and the statistical and practical significance of group differences in motor fitness capabilities. Post-menarche girls outperformed the pre-menarche girls in four of the six motor tests during baseline (T1), including EUBS, HEC, 0–10 m and 0.40 speed (Table 3). However, only the difference of 0.59 m in EUBS was of statistically significance (*p* = 0.01, *d* = 0.80). All other differences were only of practical significance in HEC (catches = 0.76, *d* = 0.17), 0.10 m speed (0.05 s, *d* = 0.27) and 0–40 m speed (0.17 s, *d* = 0.27) (Table 3).

At T2, the, post-menarche group still displayed slightly higher mean values in EUBS, HEC, 0–10 m and 0–40 m speed (*p* > 0.05) with pre-menarche having a slight advantage in ELS (*p* > 0.05) (Table 3). No difference was evident in the agility scores of the groups (Table 3). Differences between the groups also declined (*p* > 0.05) in EUBS (0.12 m), ELS (0.67 cm), agility (0.00 s) and 0–40 m speed (0.14 s), although it increased in HEC and 0–10 m speed. These differences between the groups at T2 were only of small practical significance for 0.10 m speed (*d* = 0.28), 0–40 m speed (*d* = 0.21) and HEC (*d* = 0.39) (Table 3).

At T3, no statistically significant group differences were evident, although the differences in the strength tests, EUBS (*d* = 0.36) and ELS (*d* = 0.36), showed small practical significance (Table 3). Post-menarche girls caught up and surpassed the pre-menarche group in ELS where the difference of 2.54 cm (T3) between the groups also increased from 0.67 cm during T2 to T3 (*p* > 0.05, *d* = 0.38) (Table 3). The post-menarche group still showed more favorable EUBS where between groups differences increased with 0.26 cm from T2 to T3 (*p* > 0.05, *d* = 0.36) (Table 3). The post-menarche group also showed a slight advantage in 0–10 m speed as the between-group difference of 0.01 s decreased further to similar values from T2 to T3 (*p* > 0.05, Table 3). The pre-menarche group, however, caught up and surpassed the post-menarche group during T3 in HEC (+0.15 catches) and 0–40 m (−0.05 s) speed although these both these differences were insignificant on a practical (*d* < 0.2) and statistical level (*p* > 0.05) insignificant. The pre-menarche group still showed slightly lower agility values although the difference of 0.09 s between groups increased slightly from T2 to T3, although still statistically insignificant (Table 3). Maturity group differences over time were insignificant for all motor skills (*p* > 0.05) (Table 3).

Table 4 reports the significance of changes in motor capabilities between measurements. Although various changes between measurements were of statistical significance, only changes in explosive upper body strength and 0–40 m speed in both groups and explosive leg strength in the post-menarche group were of significance from T1–T3 (Table 4). This contributed to evidence portraying that the pre-menarche group catching up and surpassing the post-menarche group in HEC and 0–40 m speed. Their larger increase in EUBS could not, however, result in them catching up to the post-menarche group (Table 3). Larger changes in ELS of post-menarche girls contributed to them surpassing pre-menarche girls during T3 although they could not catch up with pre-menarche girls in agility after showing a bigger increase as well (Table 4). Overall, the means in the different tests over this follow-up period portray that the pre-menarche groups’ motor performance, was lagging that of the post-menarche group with approximately two years. This is evident from the similar values that the pre-menarche group obtained at T3 compared to the values that were obtained by the post-menarche group at T1.

Table 5 presents the results of interaction effect between the groups over the three time points (T1–T3) derived from a repeated measures ANOVA. Interaction effects over time points were only significant for explosive upper body strength [F (2.112) = 5.9670, *p* = 0.00345], although marginally significant for explosive leg strength [F (2.108) = 2.7179, *p* = 0.06986] (Table 5).

## 4. Discussion

The objective of this study was to determine if differences exist in explosive power, speed, agility and hand-eye coordination of girls with differing menarcheal status over a two-year follow-up period. This aim was investigated by comparing pre (or late developing) and post-menarche (or early developing) girls longitudinally over time, based on their menarche status at baseline (February of Grade 8) when they had a mean age of 13.51 years.

Developmental differences between the pre- and post-menarche groups was found to be relatively small and mostly insignificant in speed, agility and hand-eye coordination. This finding agrees with most other studies, as reviewed by Malina et al. [11], that also reported similar findings. This finding is most probably attributable to the groups being already late in their pubertal phase with most of the participants (*n* = 45, 77%) having already reached menarche at baseline (T1) (Table 1). A large percentage (<38%) of the post-menarche group were also in their menarche phase for a considerable time as 4.4% reached menarche at 10 years, 2.2% at 11 years, 31.1% at 12 years and 60% at 13 years and only 2.2% at 14 years. Of the pre-menarche group, 9 (93%) reached menarche during Grade 9 (T2) at 14.51 years and all but one (98%) of this group before (T3) in their grade 10 year at the mean age of 15.51 years.

The post-menarche girls showed slightly more favorable results in the 10 m and 40 m speed although differences between the groups were insignificant. These differences were of small practical significance at 13.51 years (10 m and 20 m speed) and at 14.51 years for 40 m speed. The largest, although also insignificant differences (*p* > 0.05) were found in 10 m speed during T2 at a mean age of 14.51 years and in 40 m speed during T1 at a mean age of 13.51 years. These results coincide with the findings of Van den Berg et al. [22] who also found insignificant differences between 14-year-old girl tennis players of differing maturity levels in 10 m speed. Our study also showed similar non-significant differences in agility with the largest, mean differences (*p* > 0.05) found during T1, favoring the pre-menarche group. This could also be ascribed to pre-menarche girls having a higher percentage muscle mass (34.9%) and lower percentage fat mass (21.02%) compared to the post-menarche group (33.8%) as well as fat mass (25.84%) at T2 (*p* < 0.05), although differences in body composition became insignificant at T3 (Table 2). Hand-eye co-ordination showed insignificant differences of 1.91 catches at T2, favoring the post-menarche group. These small insignificant differences that were found and also mostly during the baseline measurements, could in some way be ascribed to the explained small differences in the time of reaching maturational status during baseline measurements in the groups. As most of the group have reached menarche in grade 9 at age 14.51 years, it can be assumed that almost all have already experienced PHV at the baseline measurements, as this event occurs on average one year before reaching menarche. The adolescent growth spurt in muscle strength and muscle endurance, which is directly linked to speed and agility, takes an onset approximately 0.5–1 year after reaching PHV where after a plateau is reached at around 14 years of age [11,49]. Therefore, due to the current maturational status of our group, where most of the participants have already reached menarche, which occurs on average two years after PHV, most participants, including participants from the pre-menarche group would have reached peak development in speed and agility. This might explain the small difference between the groups in speed and agility. Furthermore, with regard to body composition influences, Aberberga-Augskalne and Kemper [50] state that the biggest increase in mass occurs between 12 and 14 years, congruent to the average age of menarche. From the onset of menarche, estrogen plays an important role in the lipogenic profile of girls during the puberty phase [51]. Consequently, higher levels of estrogen results in a higher percentage body-fat and also the distribution of fat within the body changes to where a large percentage of fat is being distributed among the hips and surrounding areas. Increased fat distribution will have an effect on girls’ BMI and according to Kaplowitz et al. [52], girls with a higher BMI and fat percentage tend to be more advanced in their maturation status compared to girls with a lower BMI and fat percentage. As peak mass increases are concurrent with the onset of menarche, the BMI of pre-menarche girls’ (who are still to reach peak mass increases) were not affected as much compared to the post-menarche groups’ BMI. These changes could, therefore, be suggestive as possible explanations for the insignificant differences that were found in the speed and agility of girls of differing maturity levels where pre-menarche girls are still to be affected by mass increases, while post-menarche girls most probably have already adapted to peak mass increases.

Our results also showed signified interaction effects of differences in muscle strength between the pre- and post-menarche groups. A significant interaction effect was found in upper body strength (EUBS) (*p* = 0.003) with a marginally significant interaction effect in explosive leg strength (ELS) (*p* = 0.069) (Table 5), although group differences over time were insignificant for EUBS (F = 2.65; *p* = 0.11) and ELS (F = 0.02; *p* = 0.87). Differences in upper body strength during baseline measurements were also of statistical (*p* = 0.01) and large practical significance (*d* = 0.80), favoring post-menarche girls (Table 3). Post-menarche girls, furthermore, outperformed the pre-menarche group throughout the study, although the differences declined from T1 to T3, but remained practically significant (Table 3). With regard to explosive leg strength, post-menarche group also outperformed (*p* > 0.05, *d* = 0.38) the pre-menarche group although the pre-menarche group initially had an advantage in ELS (*p* > 0.05, *d* = 0.26). The results that still showed an increase in strength of post-menarche girls between 14.51 and 15.51 years in EUBS (*p* > 0.05) and ELS (*p* < 0.05), can possibly be ascribed to peak development of submaximal power output in girls that occurs more than a year after peak height velocity [11]. Therefore, due to a large percentage of our group who had already surpassed menarche and PHV, as PHV occurs approximately 12–24 months before menarche, the post-menarche group still had a slight strength advantage due to their advanced maturation. Furthermore, post-menarche girls, usually have broader shoulders because of earlier growth in body dimensions such as shoulder width [11], which may have aided them in producing more power to perform upper-body strength tests. To the contrary, peak mass increases take place between 12 and 14 years, congruent to the average age of menarche and, consequently, the pre-menarche group was most probably still affected by significant mass changes that result in fat deposits such as around the hips, which again moves the center of gravity point lower. As a result, they will have to displace more weight during upward jumping which in turn can affect performance in activities performed against gravitation negatively. The post-menarche girls might in turn, have most probably, already adapted to these body compositional changes.

Our results, furthermore, showed that the performance of the pre-menarche girls, in the different tests, based on the means that are reported during each year of measurement, was approximately two years behind that of the post-menarche group. In this regard, only small differences of 0.10 m (EUBS), 0.20 cm (ELS), 0.19 s (agility), 0.20 s (0–40 m speed) and 0.01 catches (HEC) were seen in the mean values of the pre-menarche group at T3 compared to the post-menarche girls’ performance during T1 (Table 3). This coincides with findings reported by Malina and co-workers [11] that showed a two-year difference in the mean onset of menarche between early (11.8 years) and late (>13.8 years) maturing girls. Furthermore, the adolescent acceleration in muscle strength and muscle endurance, that are directly linked to motor abilities, takes an onset approximately 0.5–1 year after reaching PHV [6]. This link between the last-mentioned factor is the result of similar accelerations in morphological and biochemical determinants (the proportion of various muscle vessel types) [53]. Furthermore, in the course of maturation, a variety of biological and physiological structures in the body functions at a higher level [24] and consequently, individuals of the same chronological age, but differing maturity status, will differ in terms of physical- and motor performance [22,54]. In this regard, Freitas et al. [26] report that factors including neuromuscular maturation, specific instruction, practice and sport participation will also affect motor capabilities between the ages of 11 and 14 years and that skeletal maturity (skeletal age according to chronological age) explains only a maximum of 2.8% variance in motor capabilities in girls, after corrections for stature and body mass. The hand–eye coordination that was tested in the study is based on a skill, which might be affected by experience and training which might explain the inconsistent results that were found in this test.

Due to growth and maturation differences that were leveled-out by the end of the follow-up period at a mean age of 15.51 years, where no significant differences were evident between the groups, it can be concluded that the speed, agility and coordination and also strength of pre- and post-menarche girls can be considered to be nearly alike in girls of the same chronological age at 16 years. Differences between pre- and post-menarche groups decreased to almost similar values in 5 of the 6 motor fitness capabilities that were tested. Explosive leg strength differences, however, increased from T1–T3 (13.51–15.51 years) in favor of the post-menarche group although the difference was still insignificant. This could be due to a larger muscle mass in relation to total body mass. Furthermore, although it was not analyzed and reported, the post-menarche girls could have had a better, well-established ratio between sitting height and leg-length, possibly resulting in a more efficient weight distribution/center of gravity. Lastly, because of earlier maturation, post-menarche girls could have a higher functioning neurological system that contributes to improved coordination, which pre-menarche girls have not yet reached.

This study had limitations that need to be acknowledged. All participants were recruited from one school, which make the results mainly relevant to the study population and thereby limits the generalization of the findings. However, the school had hostel facilities which represented children enrolling from various different primary schools from the surrounding area. Our final sample size was small and also did not evenly represent different racial groups and results are mostly based on data of white (Caucasian) girls. A power calculation of the group sizes did show that medium effects can be observed from the results. The late developing group was, however, already limited in numbers at baseline that would have constrained the power and thereby limited us in dividing this group any further at later time points because some changed to full menarcheal status at T2 and T3 which might have added further understanding of differences. Changes in environmental conditions such as weather conditions (rain, hot conditions) over the 3-year period were out of the researchers’ control and could have influenced the results. Although the data were collected over a longitudinal period of two years, which strengthens the results of the study, it has to be kept in mind that the study only incorporated secondary school learners and could subsequently only focused on a late age period in the development of girls which was between 13 and 16 years of age. It is acknowledged that a larger age period is needed to fully investigate the influences that are associated with changes in pubertal status. Although groups would have become too small for comparison purposes in the current study, it can be considered in future studies to only include girls who are the furthest in the menarche phase (post-menarche) group and those who are the furthest away from reaching menarche (pre-menarche), to make a more realistic comparison of early and late development differences. It is also advisable that influences of sport participation should also be taken into consideration in future analyses of this nature.

## 5. Conclusions

The findings of this study add to existing knowledge and provided more insight into the effect of biological and physiological changes that occur in girls before and after the onset of menarche, more specifically how these changes influence their motor fitness capabilities. The results confirm that, although there were differences in the motor capabilities of girls of differing maturity status, based on their menarche status during mid to late adolescence, these differences were only of statistical and practical significance in strength. Differences were, however, of small practical significance in agility and speed at the mean age of 13.51 years. Differences were also larger at a younger age (13.51 years) but declined with increased age. Performance in the motor tests of pre-menarche girls, were approximately two years behind that of the post-menarche girls. Based on these findings, it is concluded that the motor capabilities of girls, once they have reached menarche, will only be comparable with other girls of the same chronological age who have reached menarche earlier, 1–2 years later when the influences of puberty on their fitness performance can be considered to have been levelled-out. This knowledge can be incorporated by coaches into designing well-planned training programs, which again can aid in optimal skill development and reduce the risk of injury and drop-out. It can also contribute to improved sports development and talent identification processes, by providing more understanding of the temporary weaknesses in speed, agility and hand-eye coordination and gains in strength associated with early or late development and by applying this knowledge in appropriate short and long-term motor performance goals. Further research is, however, recommended that includes a longer developmental period, especially from a younger age. This will aid in obtaining a deeper and more complete understanding of growth differences between early and late developing girls and, consequently, the effect of differential growth on girls of different maturation’s motor capability profiles. Our study should be seen as a pilot study that can serve as a reference study and it is suggested that the findings can be used for direction and new topics when studies of this nature is planned and conducted in the future.

## Figures and Tables

**Table 1:** Descriptive characteristics of the pre- and post-menarche groups.

	Year 1 (T1)		Year 2 (T2)		Year 3 (T3)	
	N	Mean Age ± SD	N	Mean Age ± SD	N	Mean Age ± SD
Group	58	(T1) 13.51 ± 3.5	58	(T2) 14.51 ± 3.51	58	(T3) 15.51 ± 3.51
Pre-menarche	13	(T1) 13.52 ± 3.58	13	(T2) 14.52 ± 3.58	13	(T3) 15.52 ± 3.58
Post-menarche	45	(T1) 13.51 ± 3.53	45	(T2) 14.51 ± 3.53	45	(T3) 15.51 ± 3.53

T1 = Year one (Gr8); T2 = Year 2 (Grade 9); T3 = Year 3 (Grade 10); N = number of subjects.

**Table 2:** Body composition differences between pre- and post-menarche groups during T2 and T3.

	Pre	Post	Difference	*p*-Value	*d*-Value
**T1 (Grade 8)**					
BMI ∆	19.59 + 3.50	21.79 + 3.73	2.2	0.06 ^►^	0.60 ^##^
**T2 (Grade 9)**					
Fat %	21.02 ± 7.62	25.84 ± 6.45	4.82	0.02 *	0.68 ^##^
Muscle %	34.90 ± 2.63	33.38 ± 2.33	1.25	−2.02	0.61 ^##^
BMI	21.02 ± 4.07	22.36 ± 3.36	1.34	0.23	0.35 ^#^
**T3 (Grade 10)**					
Fat %	26.26 ± 10.01	28.97 ± 7.54	2.71	0.29	0.31 ^#^
Muscle %	40.24 ± 5.68	38.98 ± 4.25	1.26	0.39	0.25 ^#^
BMI	21.65 ± 3.78	22.49 ± 2.91	0.84	0.39	0.25 ^#^

T1 = Baseline measurements (Grade 8); T2 = First follow up measurement (Grade 9); T3 = Second follow up measurement (Grade 10); Pre = Pre-menarche; Post = Post menarche; * = Statistical significance (*p* < 0.05); ^►^ = Borderline significant 0.05 < *p* > 0.07; ^#^ = small effect size (*d* > 0.2); ^##^ = medium effect size (*d* > 0.5); ^###^ = large effect size (*d* > 0.8). Note ∆ BMI at T1 was calculated from mass and stature.

**Table 3:** Motor fitness characteristics and differences between the pre- and post-menarche groups at T1, T2 and T3.

	Pre-Menarche (*n* = 13)			Post-Menarche (*n* = 45)			Significance of Differences		
TP	M ± SD	Min	Max	M ± SD	Min	Max	Diff	*p*-Value	*d*-Value
Explosive upper body strength (EUBS) (m) (Maturity group * Time F = 2.65; *p* = 0.11)									
T1	4.65 ± 0.83	3.1	6.18	5.24 ± 0.63	4.05	6.72	0.59	0.01 *	0.80 ^###^
T2	5.42 ± 0.97	4.2	7.2	5.58 ± 0.57	4.5	7	0.12	0.58	0.15
T3	5.34 ± 0.80	4.04	6.73	5.60 ± 0.63	4.2	7.1	0.26	0.23	0.36 ^#^
Explosive leg strength (ELS) (cm) (Maturity group * Time F = 0.02; *p* = 0.87)									
T1	32.57 ± 4.16	26	39	31.23 ± 5.77	18.5	45	1.34	0.43	0.26 ^#^
T2	29.80 ± 8.60	19	42.5	29.13 ± 7.96	16.5	51.5	0.67	0.79	0.08
T3	31.43 ± 6.98	21	41.5	33.97 ± 6.06	22	48.2	2.54	0.2	0.38 ^#^
Agility (s) (Maturity group * Time F = 0.06; *p* = 0.80)									
T1	20.46 ± 1.22	18.57	22.43	20.72 ± 1.27	18.02	24.88	0.26	0.52	0.20 ^#^
T2	19.23 ± 1.35	17.87	22.91	19.23 ± 1.86	16.37	24.71	0	0.99	0
T3	20.53 ± 1.31	18.24	22.32	20.62 ± 1.28	18.84	24.27	0.09	0.83	0.06
Speed (0–10 m) (s) (Maturity group * Time F = 0.53; *p* = 0.47)									
T1	2.18 ± 0.15	1.92	2.45	2.13 ± 0.14	1.9	2.54	0.05	0.3	0.34 ^#^
T2	3.67 ± 0.62	1.9	4.28	3.50 ± 0.59	1.78	4.34	0.16	0.42	0.28 ^#^
T3	2.13 ± 0.15	1.88	2.39	2.12 ± 0.11	1.9	2.41	0.01	0.79	0.07
Speed (0–40 m) (s) (Maturity group * Time F = 0.04; *p* = 0.84)									
T1	7.12 ± 0.61	6.17	8.39	6.95 ± 0.64	5.81	8.9	0.17	0.39	0.27 ^#^
T2	6.88 ± 0.69	5.87	8.04	6.74 ± 0.63	5.67	8.61	0.14	0.5	0.21 ^#^
T3	6.75 ± 0.70	5.84	8.15	6.80 ± 0.63	6	8.59	0.05	0.82	0.07
Hand-eye coordination (HEC) (n) (Maturity group * Time F = 0.41; *p* = 0.52)									
T1	4.84 ± 4.45	0	14	5.60 ± 4.08	0	15	0.76	0.56	0.17
T2	6.15 ± 4.68	0	14	8.06 ± 5.02	0	20	1.91	0.22	0.39 ^#^
T3	5.61 ± 4.53	0	12	5.46 ± 4.02	0	16	0.15	0.88	0.04

TP = Time points; T1 = Baseline measurements (Grade 8), T2 = First follow up measurement (Grade 9); T3 = Second follow up measurement (Grade 10); Pre = Pre-menarche; Post = Post menarche; M = Mean values; SD = Standard deviation; * = Statistical significance (*p* < 0.05); Practical significance ^#^ = small effect size (*d* > 0.2); ^##^ = medium effect size (*d* > 0.5); ^###^ = large effect size (*d* > 0.8).

**Table 4:** Significance of changes in motor fitness characteristics of pre- and post-menarche groups.

	T1–T2		T2–T3		T1–T3	
	Pre	Post	Pre	Post	Pre	Post
Explosive upper body strength	0.81 *	0.34 *	−0.12	0.02	0.69 *	0.36 *
Explosive leg strength	−2.77	−1.9	1.63	4.75 *	−1.14	2.85 *
Agility	−1.23 *	−1.38 *	1.30 *	1.24 *	0.07	−0.14
Speed (1–10 m)	1.42 *	1.36 *	−1.45 *	−1.38 *	−0.03	−0.02
Speed (0–40 m)	−0.23 *	−0.17 *	−0.04	0	−0.27 *	−0.17 *
Hand-eye coordination	1.31	2.46 *	−0.54	−2.64 *	0.77	−0.18

* = Statistical significance (*p* < 0.05); T1 = Baseline measurements (Grade 8), T2 = First follow-up measurement (Grade 9); T3 = Second follow-up measurement (Grade 10); Pre = Pre-menarche; Post = Post-menarche.

**Table 5:** Interaction effects between pre- and post-menarche groups over time of.

Variable	Interaction Effect
Explosive upper body strength	F (2.112) = 5.9670, *p* = 0.00345
Hand-eye coordination	F (2.112) = 2.2069, *p* = 0.11480
Vertical jump	F (2.108) = 2.7179, *p* = 0.06986
Agility	F (2.98) = 0.12702, *p* = 0.88086
Speed 0–10 m	F (2.92) = 0.10077, *p* = 0.90424
Speed 0– 40 m	F (2.100) = 0.54467, *p* = 0.58174

*p* = statistical significance set at *p* < 0.05.

## Data Availability

The dataset is the property of the North-West University under supervision of Prof Anita E Pienaar. In this regard, Prof. AE Pienaar should be contacted if, for any reason, the data included in this paper needs to be shared.
